# Improved Impact Properties in Poly(lactic acid) (PLA) Blends Containing Cellulose Acetate (CA) Prepared by Reactive Extrusion

**DOI:** 10.3390/ma12020270

**Published:** 2019-01-15

**Authors:** Maria-Beatrice Coltelli, Norma Mallegni, Sara Rizzo, Patrizia Cinelli, Andrea Lazzeri

**Affiliations:** 1 Department of Civil and Industrial Engineering, University of Pisa, Via Diotisalvi, 2, 56126 Pisa, Italy; norma.mallegni@gmail.com (N.M.); sararizzo@outlook.com (S.R.); patrizia.cinelli@unipi.it (P.C.); 2 National Interuniversity Consortium of Materials Science and Technology (INSTM), c/o Via Diotisalvi, 2, 56126 Pisa, Italy

**Keywords:** poly(lactic acid), reactive extrusion, cellulose acetate, impact properties, phase morphology

## Abstract

Poly(lactic acid)/triacetine plasticized cellulose acetate (PLA/pCA) blends were prepared by extrusion at two different temperatures and tetrabutylammonium tetraphenyl borate (TBATPB) was added as a transesterification catalyst to reactively promote the formation of PLA-CA copolymer during the reactive extrusion. The occurrence of chain scission in the PLA phase and branching/crosslinking in the CA phase in the presence of TBATPB, resulting also in a darkening of the material, were demonstrated by studying torque measurements and by performing proper thermogravimetric tests on CA with the different additives. Tensile and impact tests onto the blends prepared at the lower temperature showed better properties than the ones obtained at a higher temperature. Then, the mechanical properties of PLA/plasticized cellulose acetate (pCA) blends prepared at the lower temperature were investigated as a function of the content of plasticized CA in the blend. A range of compositions was observed where blends exhibited improved impact properties with respect to pure PLA without a significant decrease in their elastic modulus. The study of the phase morphology of the blends revealed that the occurrence of reactive compatibilization did not significantly affect the phase distribution. In general, fibrillar CA particles were formed in the PLA matrix during extrusion, thus allowing the preparation of CA fibre reinforced composites. The trend of morphology as a function of the composition and processing conditions was then discussed by considering the evolution of phase morphology in immiscible polymer blends.

## 1. Introduction

Polylactide (PLA), a polymer derived from lactic acid (2-hydroxy propionic acid), is currently proposed in many applications because it can be obtained from renewable agricultural sources [1,2], its production consumes carbon dioxide [3], it provides significant energy savings [4], it is recyclable and compostable [5], it can promote the growth of agricultural activities and its physical and mechanical properties can be modulated by properly designing the polymer architecture [6,7]. The use of PLA is thus advantageous in view of the application of circular economy principles, but some improvements in its performance are still necessary to allow its use in specific sectors. In particular, the improvement of mechanical resistance at high temperatures and of toughness are fundamental goals for PLA-based materials. As its glass transition temperature is at about 60 °C, applications requiring maintenance of the stiffness at higher temperatures, such as in rigid packaging for hot beverages [8] or in the automotive industry [9], cannot occur with sufficient reliability, thus limiting the application field of PLA. Moreover, the impact properties are poor and necessitate improvement [10] by maintaining a high stiffness. Several approaches have been considered in the literature to overcome these issues, and this topic is the object of a recent review by Nagarajan et al. [11].

In order to improve the mechanical resistance at high temperatures, three main approaches have been followed up until now: branching/crosslinking, the production of composites and the production of blends. 

The branching/cross-linking of PLA with proper chain extender/branching agents is an example [12,13,14,15]. Thanks to the characterization by Dynamical Mechanical Thermal Analysis (DMTA), it was demonstrated that the tan δ peak of PLA shifts to a higher temperature by increasing the amount of dicumyl peroxide added to PLA [13]. However, by using peroxides [12], because of the occurrence of many side reactions, the viscosity in the melt is also modified. Hence, the applicability of this method requires better control of the processing rheology necessary for the final application, and the effect on impact properties is very limited.

Another approach followed for improving the mechanical resistance at high temperatures is the preparation of composites or nano-composites in which proper fibres or particulate fillers can reinforce the PLA matrix. Mineral fillers such as phyllosilicates [16,17,18], halloysite [19], sepiolite [20], graphene [21] or carbon nanotubes [22,23] with different shapes and aspect ratios were considered, and overall, a good reinforcing effect of these fillers was evidenced, although their behavior is often complicated by their effect on PLA crystallization [24]. Moreover, many papers have been published on PLA-based composites reinforced with fibres, mainly cellulosic [25,26] or nano-cellulosic fibres [27]. The use of fibres is often responsible for changes in processing conditions, but a good balance of stiffness and impact resistance is usually achieved. The improvement in stiffness is attributed to the interactions between filler surfaces and the polymer matrix, which act as physical cross-linking points in the system. The improvement of impact properties typical of these composites is ascribed to the pull-out mechanism, by which the exit of the fibres from the polymer matrix during the impact can provide a mechanism to dissipate the impact energy. Thus, the impact strength of the material is improved.

Another interesting strategy for improving PLA mechanical resistance at high temperatures is blending with polymers that have a glass transition temperature higher than that of PLA [28]. In particular, blending with poly(carbonate) of bisphenol A, which has a glass transition temperature of about 150 °C, has been investigated by several researchers [10,29,30,31,32]. The extrusion of poly(lactic acid)/polycarbonate (PLA/PC) blends in the presence of a catalyst consisting of triacetine and tetrabutylammonium tetra-phenylborate (TBATPB) was successful in achieving this result [31,33]. Dynamic mechanical thermal analysis revealed a new peak attributable to the glass transition temperature of the PLA-PC copolymer at a temperature lower than the T_g_ typical of PC and higher than the T_g_ of PLA. Thanks to this new transition, the thermal stability of the material was improved, and also, the elastic modulus of the blends improved. However, these blends had the disadvantage of being not completely biodegradable [31] because of the presence of the polycarbonate phase. 

Cellulose esters can be obtained industrially by the esterification of cellulose [34]. Cellulose acetate (CA) is the most important cellulose derivative, and it can be used in a wide range of applications [35]. It has a glass transition temperature of about 200 °C. Hence, it is a good candidate to be blended with PLA for the improvement of both toughness and mechanical resistance at high temperatures. Regarding its renewability, CA can be synthesized by acetylation of either all or partial hydroxyl groups of cellulose. In terms of dependence on the degree of acetylation, it was demonstrated to have a different biodegradation behavior [36]. Cellulose diacetate (CDA), having an average acetylation degree of about 2.1–2.5, is biodegradable [37]. However, its processing window is very narrow due to the close proximity between its flow temperature and decomposition temperature, which limits the processability and extensive application of the material [38,39]. The plasticization of CA in a discontinuous mixer, in a simple shear regime, or in solutions with different renewable plasticizers was reported to be a successful strategy for modulating its properties [40,41]. In particular, Phuong et al. [42] demonstrated that the plasticization of cellulose diacetate with triacetine at up to at least 20% of its weight was successful for obtaining a material that was easy to extrude. In good agreement, Zepnik et al. [43] used triethyl citrate to modulate the elongational flow properties of cellulose acetate.

Only a few papers have been published up until now about the blending of PLA and CA. Ogata et al. [44] prepared poly(lactic acid)/cellulose diacetate films by solution casting and noticed that the micro-mechanical properties of the blends could be modulated as a function of composition. In particular, the DMTA studies showed that the addition of CA to PLA effectively allowed the preservation of a consistent resistance above PLA glass transition. The use of titanium isopropoxide as a transesterification catalyst seemed to be effective for improving the homogeneity of the films. 

Wang et al. [45] grafted maleic anhydride on PLA in solution, achieving a functionalization degree of 1.13 wt%. Then, after purification, they used pure PLA, or the grafted PLA for comparison, to prepare PLA/cellulose acetate blends of various compositions with PLA as the main component in a discontinuous mixer at 160 °C, hence below the melting temperature of CA. They did not observe an improvement in properties with respect to pure PLA, but they observed a significant increase in tensile strength in blends containing grafted PLA with respect to those containing PLA. On the contrary, in the latter the addition of CA to PLA resulted in a significant decrease in tensile strength, going from 45 MPa for pure PLA to about 20 MPa for the blend at 20% of CA.

More recently, Quintana et al. [46] prepared PLA/triacetine plasticized CA in an internal mixer. They provided evidence that the two polymers are immiscible and prepared compatibilizers for the blend by grafting PLA chains on cellulose acetate by ring opening polymerization of L-lactide, D-lactide or mixtures of them [47,48]. They observed an improvement in adhesion between the two phases and an improvement in properties in some compatibilized blends, suggesting, in agreement with Ogata [44] and Wang [45], the necessity of improving the compatibility of the blend.

In the present work, the preparation of PLA/plasticized cellulose acetate (pCA) blends is, for the first time, performed by extrusion, hence, in the presence of an elongational flow that can strongly affect phase morphology evolution. The processing methodology and tensile and impact properties are investigated as a function of the pCA content in the blend. Moreover, a reactive compatibilization approach based on the use of TBATPB as a transesterification catalyst is promising, because it has been found to be successful in PLA/PC blends in our laboratory [31,32]. In particular, tensile and impact properties of the blends are discussed as a function of composition. The trend of morphology as a function of the composition and processing conditions is also discussed, with the aim of providing a correlation with final properties.

## 2. Materials and Methods 

### 2.1. Materials

Triacetine plasticized CA (pCA), with a content of triacetine of 20 wt%, and pure CA were purchased from GIBAPLAST (Varese, Italy). Poly (l-lactic) acid, purchased from NatureWorks LLC (Minnetonka, MN, USA), was Ingeo™ 2003D Extrusion Grade, having a nominal average molecular weight Mw of 199,590 and a density of 1.24 g/cm^3^. Triacetine (TA), also known as glycerin triacetate or 1,2,3-triacetoxypropane, and tetrabutylammonium tetraphenylborate (TBATPB, CAS #15522-59-5) were purchased from Aldrich Chemicals (Saint Louis, MO, USA). 

### 2.2. Processing

The PLA and pCA were dried for 24 h at 60 °C in a ventilated oven before extrusion. The PLA/pCA blends that were eventually pre-mixed with the TBATPB in powder, were extruded in a MiniLab II Haake™ Rheomex CTW 5 conical twin-screw extruder (Thermo Fisher Scientific, Waltham, MA, USA) at 230 °C and at 197 °C. The screw rate was 100 rpm, and the duration of extrusion was 60 s. After extrusion, the molten materials were transferred through a preheated cylinder to the Haake™ MiniJet II mini injection moulder (Thermo Fisher Scientific, Waltham, MA, USA), to obtain Haake type-III specimens (total length 87 mm, total width 10 mm, thickness 1.5 mm) that were used for tensile measurements and ISO 179 specimens for Charpy Impact tests. 

### 2.3. Characterization

Torque measurements were performed by using the MiniLab II Haake™ Rheomex CTW 5 conical twin-screw extruder (Thermo Fisher Scientific, Waltham, MA, USA) at 230 °C and at 197 °C. The extrusion was monitored for up to 180 s to obtain the trend as a function of the extrusion time. The measurements were carried out three times, and the average value and standard deviation were calculated for each trend.

Charpy Impact tests on Notched specimens were carried out by using a CEAST 9050 (CEAST, Torino, Italy), equipped with DAS 8000 Junior (CEAST, Torino, Italy) for data recording at a frequency of 1000 kHz following the ISO 179 standard. 

Tensile tests were performed at room temperature at a crosshead speed of 10 mm/min by means of an Instron 4302 universal testing machine (Canton, MA, USA) equipped with a 10 kN load cell and interfaced with a computer running the TestWorks 4.0 software (MTS Systems Corporation, Eden Prairie, MN, USA).

The morphology of the blends was studied by scanning electron microscopy (SEM) using a JEOL JSM-5600LV (Tokyo, Japan), by analysing cryofractured sample surfaces that had been previously sputtered with gold.

Thermogravimetric tests (TGA) were carried out with a Rheometric Scientific Instrument (New Castle, DE, USA) balance using samples of about 10 mg. CA samples consisted of powder of pure CA. The content of TBATPB was 5%, and the content of triacetine was 20%. The two additives were thoroughly mixed with the CA powder before the TGA analysis. Tests were made in nitrogen (60 mL⋅min^−1^), in a temperature range between 30 and 1000 °C, with a scanning rate of 10 °C/min.

Dissolution tests aimed at studying the phase morphology evolution in the blends by integrating with SEM results were carried out by using a mixture of acetone and water—90/10 by weight. In fact, by performing a preliminary study, it was found that this mixture is selective for the dissolution of the CA. A piece of extruded strand (extrusion time of 60 s) of each blend was weighed and put in acetone/water for 24 h by stirring. After washing with fresh solvent, the residue was dried in an oven at 60 °C for 24 h and weighed. 

## 3. Results and Discussion

### 3.1. Effect of Extrusion Parameters on Reactivity and Properties of the Blends

In order to study the effect of extrusion temperature and TBATPB transesterification catalyst on the PLA/pCA blends extrusion, four preliminary blends with a content of PLA of 75% by weight were prepared (Table 1). 

**Figure 1:**
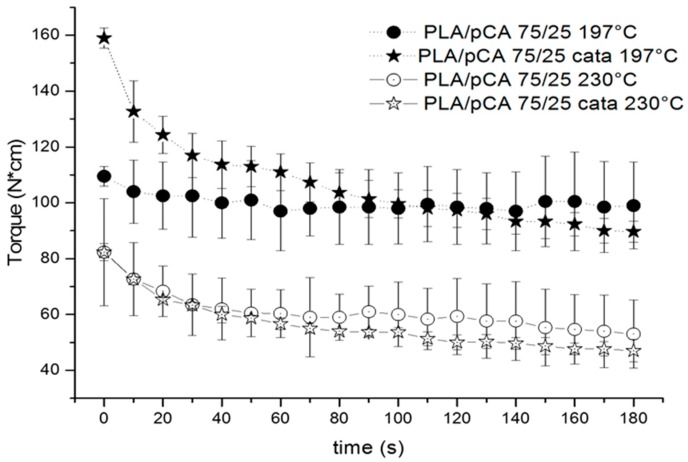
Trends of torque as a function of time recorded for PLA/pCA blends prepared at 230 °C and 197 °C with or without TBATPB. N*cm = N·cm.

It can be noticed in Table 1 by examining torque values, or more clearly in Figure 1, that the torque at 180 s depends essentially on the selected temperature. By decreasing the temperature from 230 °C to 197 °C, doubling of the torque value is obtained. The TBATPB addition caused only a slight decrease in torque, probably due to chain scission occurring during extrusion that is absolutely negligible at 60 s (extrusion time). The decrease in extrusion temperature below 197 °C leads to torque values that are quite high. Moreover, the recovery and injection moulding of material for preparing specimens was difficult because the viscosity was much too high.

**Figure 2:**
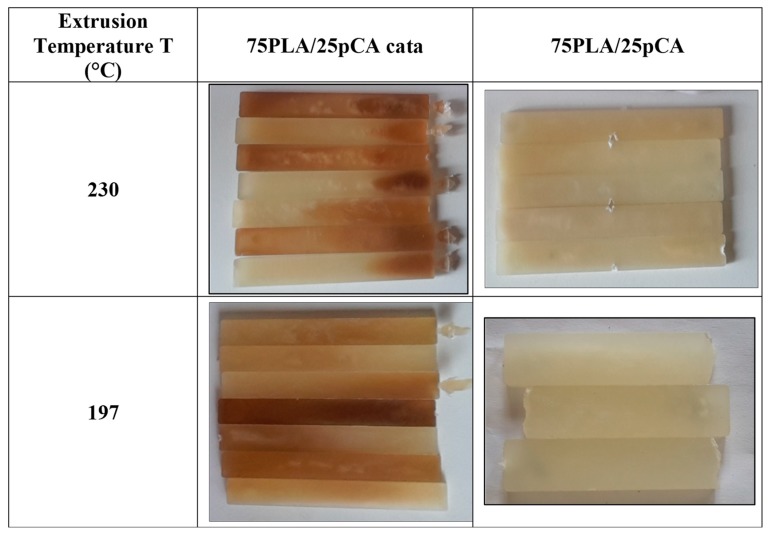
Specimens’ color after extrusion and injection moulding.

The trends of torque as a function of time were almost constant during extrusion in the prepared blends, in agreement with the limited impact on the melt viscosity of undesired reactions, such PLA hydrolysis. However, the trials carried out with the TBATPB catalyst resulted in evident specimen darkening (Figure 2). The darkening was more evident at the higher temperature.

**Figure 3:**
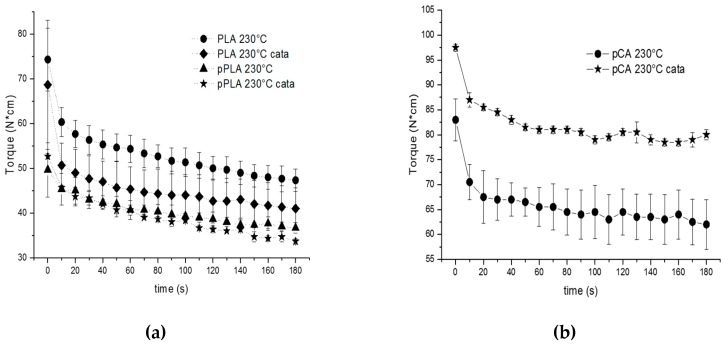
Torque as a function of time: (**a**) for the blends prepared with pure PLA-based samples; (**b**) for the blends prepared with pCA-based samples. N*cm = N·cm.

To explain the observed results, it was necessary to carry out some trials, which are listed in Table 2, on pure polymers by extruding the different additives one by one with PLA. The trials were carried out at 230 °C, as this temperature is the one between those adopted that is suitable for the processing of pCA alone. As is evident by the data in Table 2, the addition of triacetine to PLA resulted in a decrease in the torque due to the decreased melt viscosity. The addition of TBATPB to pure PLA (run PLA cata) determined a slight but significant decrease in the torque at 180 s, suggesting the occurring of chain scission (due to humidity) catalysed by TBATPB, in agreement with its behavior as a transesterification catalyst. Despite the drying operation being carried out on the material before extrusion, the occurrence of chain scission can be attributed to the residual humidity in the material and to the environmental humidity present during the introduction of the material into the extruder. The addition to PLA of both triacetine and TBATPB resulted in a further decrease in the torque value at 180 s. The trend of the torque as a function of time can be observed in Figure 3a. It is possible to notice that in the absence of triacetine, the decrease in torque due to the addition of the catalyst was higher than in its presence. The result can be justified considering that the catalyst interacts preferentially with triacetine, being the most abundant and mobile chemical species. However, the triacetine, which contributes to PLA chain scission, also acts as a branching molecule. Thus, the decrease in torque due to chain scission is partly counterbalanced by the branching action of the triacetine, as previously explained in the work by Phuong et al. [31].

Interestingly, the trials performed on plasticized CA involving the addition of TBATPB showed a significant increase in the torque value and also the trends as a function of time (Figure 3b) confirmed this significant melt viscosity change. The change can be attributed to the branching/cross-linking reaction occurring on cellulose acetate.

**Figure 4:**
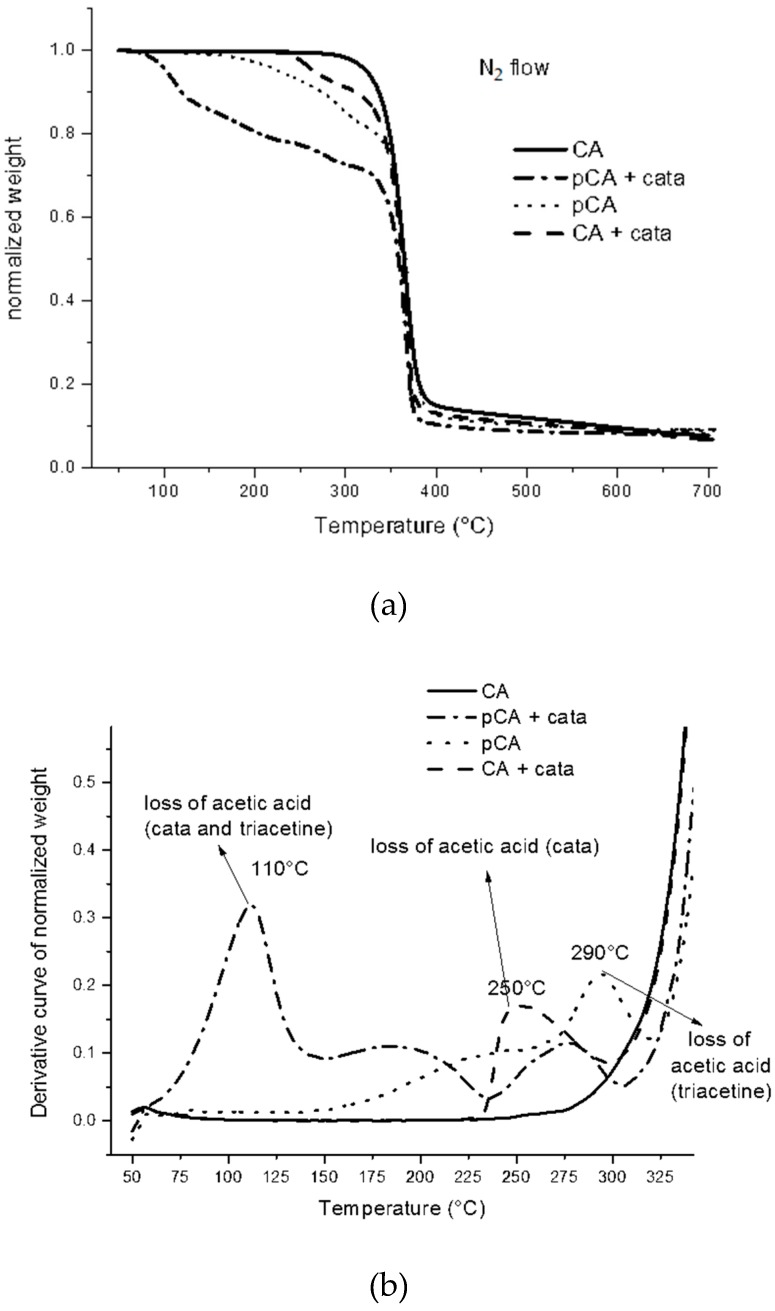
Thermogravimetry experiments on CA samples: (**a**) mass loss as a function of temperature; (**b**) enlargement in the 50–325 °C range of the derivative curve to show mass loss inflection points.

In order to better understand this latter process, some TGA experiments were carried out in nitrogen flow onto pure CA powder, as well as on the same with TBATPB, triacetine or both the additives. The mass vs. temperature curves were elaborated to identify the temperature at which eventual mass losses could be observed. The mass losses (Figure 4a) are due, at temperatures of up to 300 °C, to the volatilization of the chemical species present or formed during the experiment. Above 300 °C, more extensive degradation involving the CA macromolecular structure occurs [49]. In the sample consisting only of TBATPB and CA, a slight variation was observed at 250 °C with respect to the trend of pure CA, likely due to the loss of acetic acid [50] and probably facilitated by the presence of the catalyst that interacts with the C=O of the acetyl groups on the CA macromolecules. In the case of plasticized CA (pCA), the mass loss begins slowly at about 200 °C and is due to the volatilization of triacetine, having a boiling point of 258 °C. A further mass loss at 290 °C can be attributed to the loss of acetic acid. Interestingly, it has been reported that this molecule results from the degradation under pyrolysis conditions of both triacetine [51] and CA [50].

It is very evident, especially by examining the derivative curve and enlarging the scale in the 50–330 °C range (Figure 4b), that the combination of triacetine and TBATPB in the sample of pCA cata had the highest mass loss and this occurred at a lower temperature (110 °C). 

Regarding the purpose of our work, these experiments show that the heating of CA at a high temperature in the presence of triacetine results in evident reactivity. This is due to the formation of more activated species from triacetine bearing hydroxyl groups, as explained in Phuong et al.’s work [31]. Inside the PLA phase, under processing conditions, they can result in chain scission and some branching [31]. In the CA phase, as nucleophiles, they can promote the formation of acetic acid from CA. In fact, the formation of acetic acid, having a boiling point of 118 °C, has been reported as typical in research works about CA thermal degradation. The degradation mechanism is based on the formation of acetic acid and a C=C double bond on the CA backbone [50].

During the extrusion, the conditions are isothermal and not quiescent; hence, they are quite different with respect to those of TGA experiments. However, in Figure 4a, it is evident that a strong mass loss was achieved in the temperature range typical of extrusion (197–230 °C). Hence, we can conclude that the cross-linking reaction observed during the extrusion can occur in combination with the loss of volatile species. Under conditions of extrusion, only a slight and partial loss of volatiles can occur because of the short time of extrusion (60 s). The cross-linking can thus probably occur through a radical mechanism involving the formation of double C=C bonds because of acetic acid loss. As these bonds, especially if conjugated, absorb visible light, the occurrence of darkening in the presence of the TBATPB of the blend can be reasonably explained. In the absence of TBATPB, the pCA is thermally stable, on the basis of the TGA experiment, up to 290 °C. Accordingly, any evident darkening is observed in samples without TBATPB.

The mechanical properties of the PLA75/pCA blends obtained at 230 °C and 197 °C were measured. The results are reported in the general figures regarding mechanical properties. The impact properties showed a significant improvement with respect to pure PLA. The improvement was more evident in the blends prepared at 197 °C than in the blends prepared at 230 °C, whereas the presence of TBATPB did not influence these properties much. The elastic modulus also improved with respect to pure PLA, with the exception of the blend obtained at 230 °C without TBATPB. The tensile strength decreased in all blends with respect to pure PLA, but the decrease was more evident in the case of the blends prepared at 230 °C. The elongation at break decreased in all blends with respect to pure PLA. The mechanical properties were, on the whole, better for the blends prepared at 197 °C than for the blends prepared at 230 °C. 

In general, the properties are not correlated to the darkening observed in the samples and depend more on temperature, suggesting an impact of the processing conditions on the blend morphology. Hence, in order to better understand the properties that are achievable thanks to this process, blends with and without TBATPB were prepared by varying the composition in terms of percentage of pCA.

### 3.2. Properties and Morphology of PLA/pCA Extruded Blends as a Function of Composition and Reactive Compatibilization

**Figure 5:**
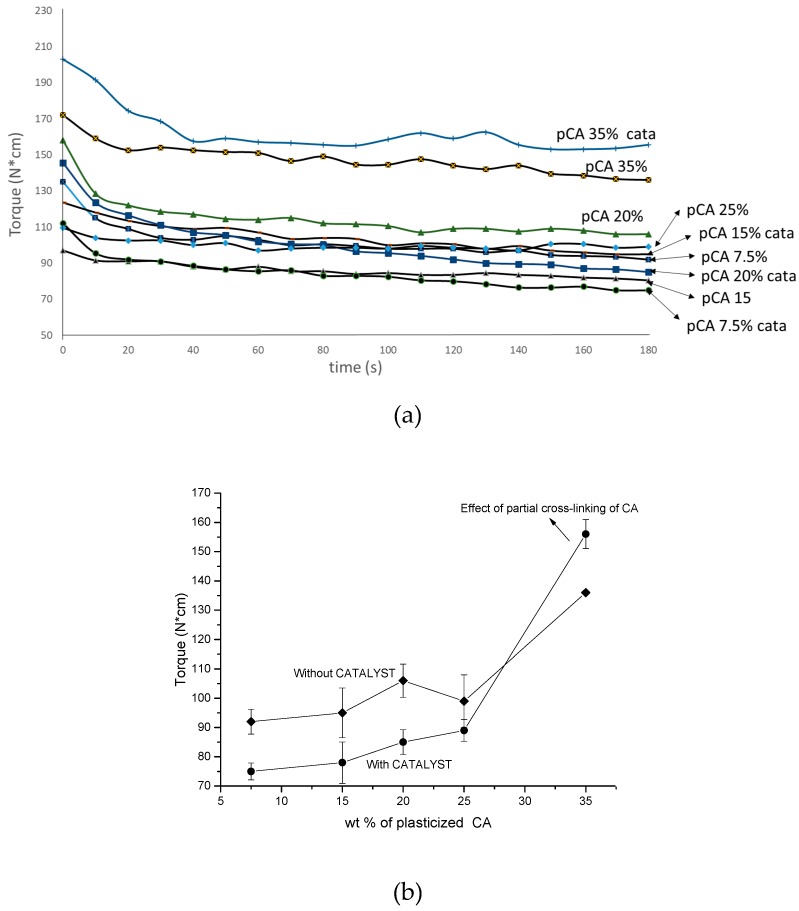
Torque measurements of blends prepared at 197 °C (**a**) as a function of time; (**b**) torque at 180 s as a function of pCA content. N*cm = N·cm.

The study of torque as a function of time for the blends prepared at 197 °C showed a general increase by increasing the content of pCA, in agreement with the observation that the viscosity of pCA was reasonably higher than the viscosity of PLA (Figure 5a,b). However, the blends containing 35% of pCA showed the highest values of torque, and a difference of about 20 N*cm was noticed with respect to the blends with a lower content of pCA. Interestingly, the trend as a function of pCA content showed lower values of torque than the uncatalysed blends when the content of pCA was lower than 30%. Probably, the predominant reaction in the presence of TBATPB in these blends is the PLA chain scission, leading to a decrease in melt viscosity. When the content of pCA is higher than 30%, the torque of the catalysed blend is higher than the uncatalysed blend. This is in agreement with a strong influence on viscosity of the cross-linking of cellulose acetate (Figure 5b).

**Figure 6:**
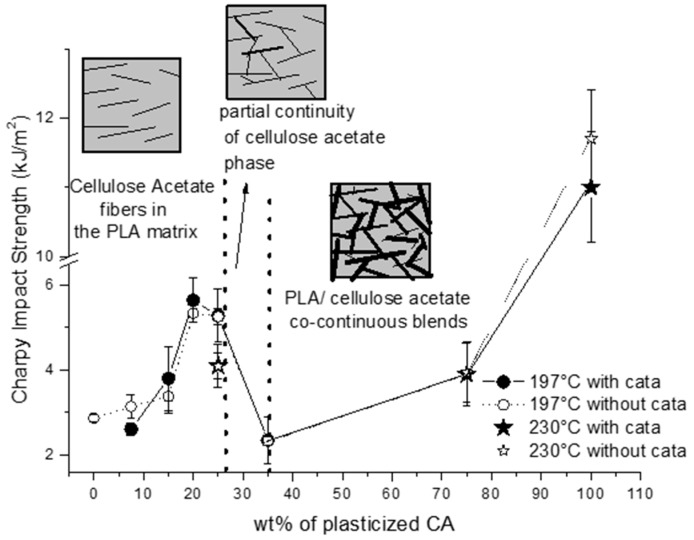
Charpy impact strength as a function of pCA content in the blends. The phase morphology as a function of pCA content in PLA/pCA blends is also indicated.

The results on the Charpy impact strength are reported in Figure 6. The trend as a function of composition showed that a significant improvement in impact properties with respect to pure PLA was achieved at a composition of 20–25% of pCA, in the presence or in the absence of TBATPB. At 35% of pCA, a decrease in the Charpy impact strength was noticed. The increase in pCA content up to 75% resulted only in a slight improvement in impact strength. It is possible to notice that the pure plasticized cellulose acetate showed an impact strength of about 12 kJ/m^2^; hence, the addition of PLA to pCA was detrimental for impact properties. On the contrary, the addition of pCA at a composition of 20–25% to PLA seems to be quite convenient by comparing its impact properties with those of pure PLA.

**Figure 7:**
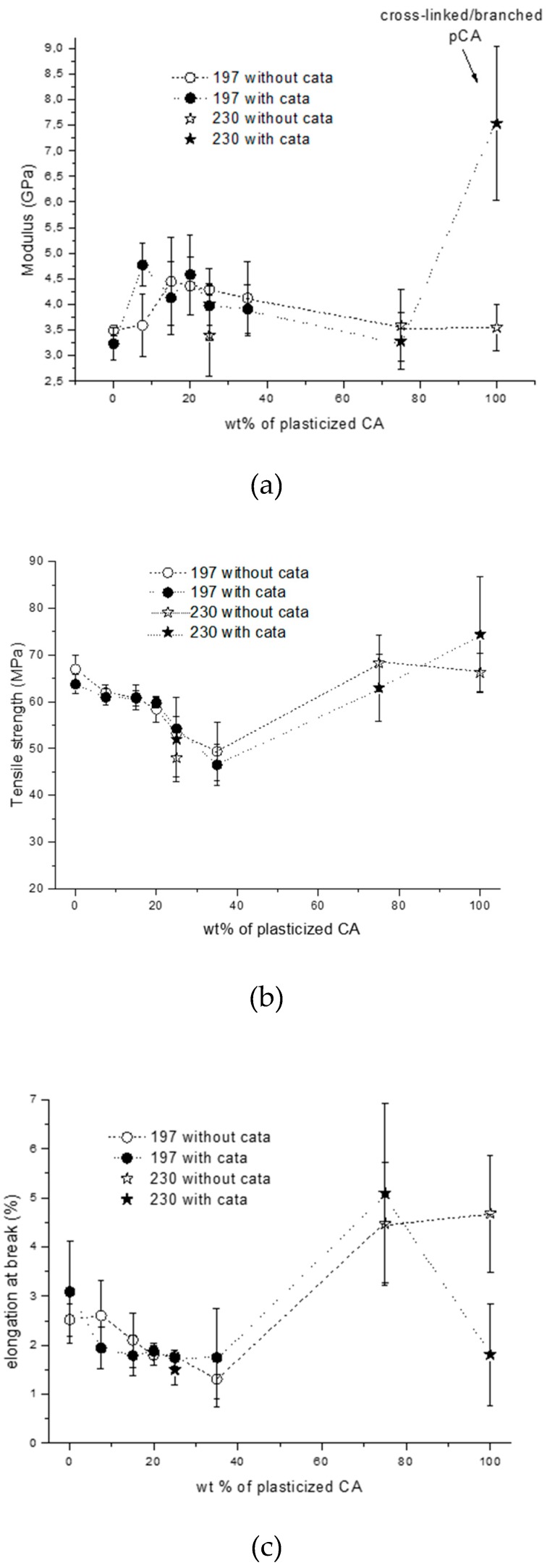
Tensile properties of blends as a function of content of pCA: (**a**) elastic modulus; (**b**) tensile strength; (**c**) elongation at break.

The tensile properties were also determined, and it was found that the addition of pCA to PLA resulted in an increase in the elastic modulus with respect to pure PLA (Figure 7a). In particular, the blends that showed improved impact strength also showed an increased elastic modulus. This represents a very good achievement. In fact, an increase of about 1 GPa (up to 4.5 GPa) was achieved with respect to pure PLA. When the content of pCA was above 35% the modulus decreased, and it further decreased for a content of pCA of 75%. However, the pure pCA showed a higher modulus with respect to pure PLA. Interestingly the modulus was highly improved in the pCA sample with TBATPB. This peculiar result can be explained by taking into account the occurrence of cross-linking in this sample, leading to an increased rigidity of the macromolecular structure.

The tensile strength slightly decreased with respect to pure PLA by adding up to 20% of pCA (Figure 7b). Above this threshold, the tensile strength decreased more, and it reached a minimum value at about 35% of pCA. Then, above this composition, the tensile strength increased, and in samples based on pCA at 75%, the tensile strength was similar to that of PLA and pCA.

The elongation at break was quite low in pure PLA, and it decreased as a function of the pCA content up to 35% (Figure 7c). Above this value, the elongation at break increased by up to about 5% for pure pCA. Interestingly, the elongation at break was significantly lower for the pCA, in agreement with the occurring of cross-linking.

**Figure 8:**
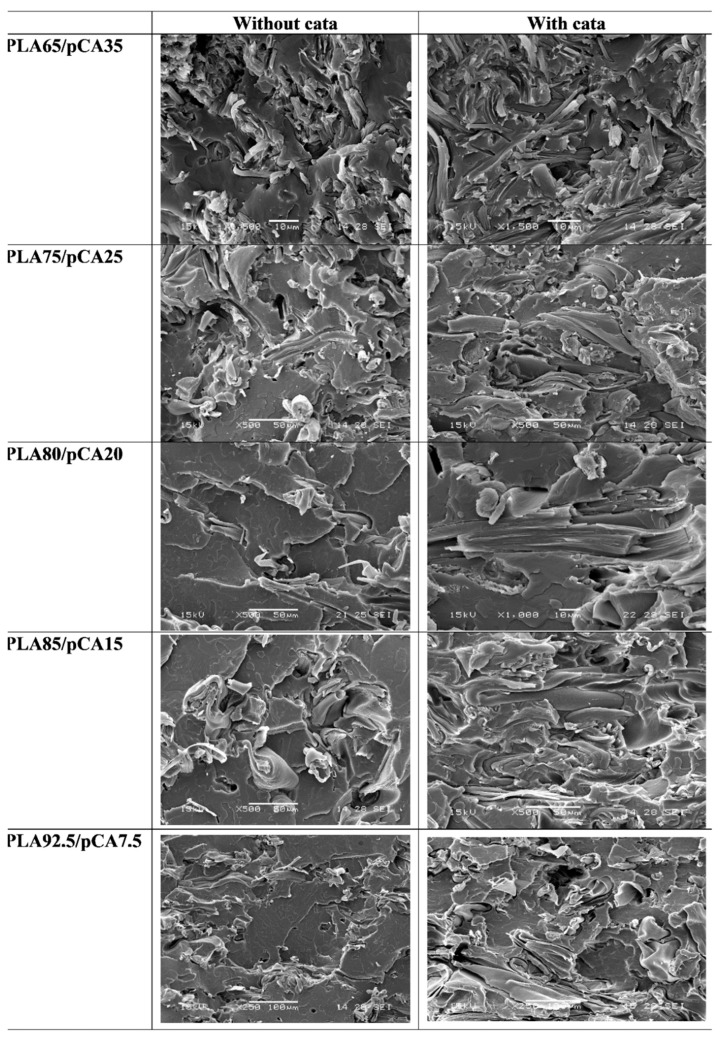
Scanning electron micrographs related to the PLA/pCA blends prepared at 197 °C with and without TBATPB.

The trends for the impact and tensile properties as a function of composition can be explained by also taking into account the phase morphology of the blends. In Figure 8, SEM micrographs are reported for each blend prepared at 197 °C. It can be noticed, starting from the bottom of the figure, that the addition of 7.5% of pCA in the PLA matrix resulted in both catalysed and uncatalysed blends in a dispersed morphology having a PLA matrix and dispersed cellulose acetate fibres with a thickness of about 10 µm. Hence, cellulose acetate reinforced composites were prepared through this extrusion process at 197 °C. A similar morphological structure can be observed also for 15% and 20% pCA blends, as a similar fibrillated structure can be observed with 10 µm thick fibres but with a higher number of fibres because of the increased pCA content. The adhesion between fibre and matrix seems high both in the uncatalysed and catalysed composites. This is very evident in the micrograph of PLA80/pCA20 cata blend (Figure 8).

**Figure 9:**
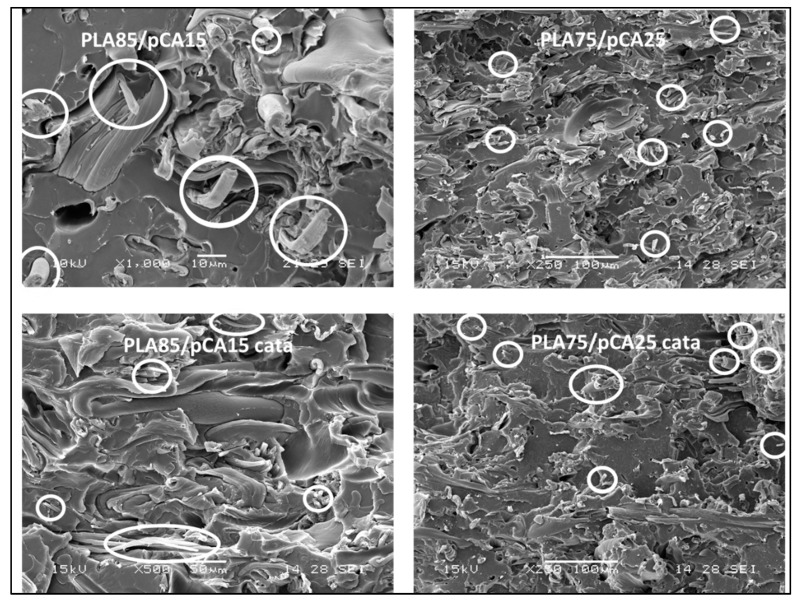
Scanning electron micrographs where single fibres are indicated by circles. The micrographs are related to the PLA/pCA 85/15 and 75/25 blends prepared at 197 °C.

To better show the presence of this fibrillar morphology, some more micrographs with respect to those of Figure 8 are reported in Figure 9, selecting PLA85/pCA15 and PLA75/pCA25 compositions as examples (with and without catalyst), where single fibres that are more visible on cryofractured surfaces are indicated by circles. This fibrous morphology was evident in all the blends prepared at 197 °C, having contents of pCA between 8 and 35%. 

The increased toughness of these composites, which was previously known and exploited by purposely adding natural or synthetic fibres to PLA [52,53], can be explained by taking into account the possibility of fibre reinforced composites to dissipate impact energy, thanks the interface energy and pull out mechanism [54].

By increasing the amount of pCA at 25%, tendencies for the interconnection of fibres and the formation of a partial co-continuous phase morphology were shown. In the blend containing 35% by weight of pCA, the interconnection of fibres seemed complete; hence, a fully co-continuous morphology was achieved.

**Figure 10:**
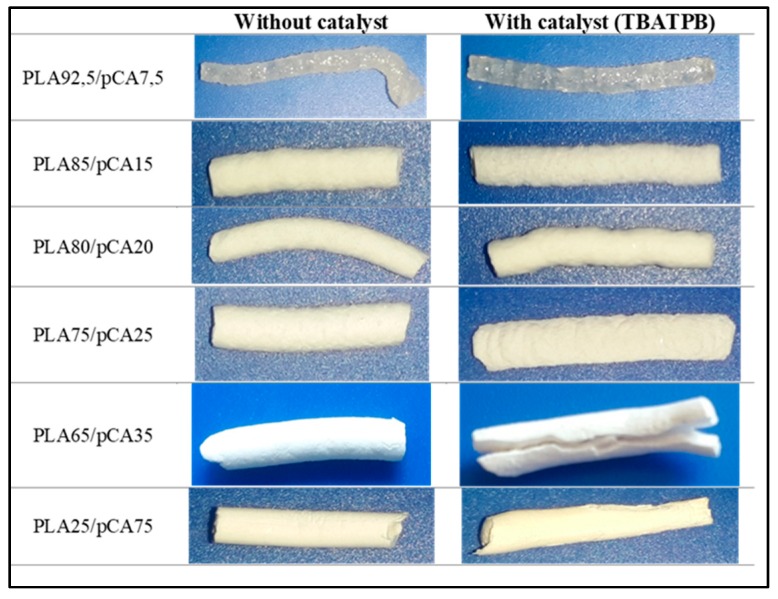
Pictures of residues after dissolution tests carried out on extruded strands of PLA/pCA blends obtained with or without TBATPB.

In order to have more information about the phase morphology evolution, dissolution tests were performed to selectively dissolve cellulose acetate onto extruded strands recovered from the extruder after 1 minute of processing. The percentage by weight of residue, consisting of the PLA undissolved phase, is reported in Table 3, and the picture of the different extruded strands after dissolution tests is reported in Figure 10. 

Interestingly, it can be noticed from the picture that all the examined blends showed a continuous PLA phase. On the other hand, examining Table 3, it is possible to understand that when the dissolution of the pCA phase is complete, it is reasonable to hypothesize the existence of a co-continuous structure. In fact, the pCA dissolved ratio can be calculated as the ratio between the percentage of extracted pCA, determined as the weight of the extracted fraction, with respect to the fed pCA. A ratio close to or higher than one indicates that the solvent can extract easily the pCA from the blend; hence, the pCA is a continuous fully interconnected phase. On the contrary, when the ratio is lower than one, it means that the pCA is dispersed in the PLA matrix, and then the solvent cannot extract it completely. In Figure 10, the ratio defined above is reported as a function of the pCA content in the blend. It is evident that above 35%, the ratio becomes very close or higher than 1, in agreement with a co-continuous morphology. 

By considering the two sets of information (SEM and dissolution tests) and also taking into account the results of torque, indicating a strong increase when the content of pCA was higher than 35%, the morphology evolution of the uncatalysed and catalysed systems were shown to be similar: the morphology was dispersed with a fibrillar dispersed phase in the PLA matrix with up to 25% of pCA; then, in between 25 and 35%, the pCA phase became partially continuous; and at 35% of pCA content, a fully co-continuous phase morphology was achieved. Above 35%, the blends were co-continuous with up to more than 75% of pCA. Hence this PLA/pCA system shows a very broad range of co-continuity and an inversion point at about 60% by weight of pCA.

**Figure 11:**
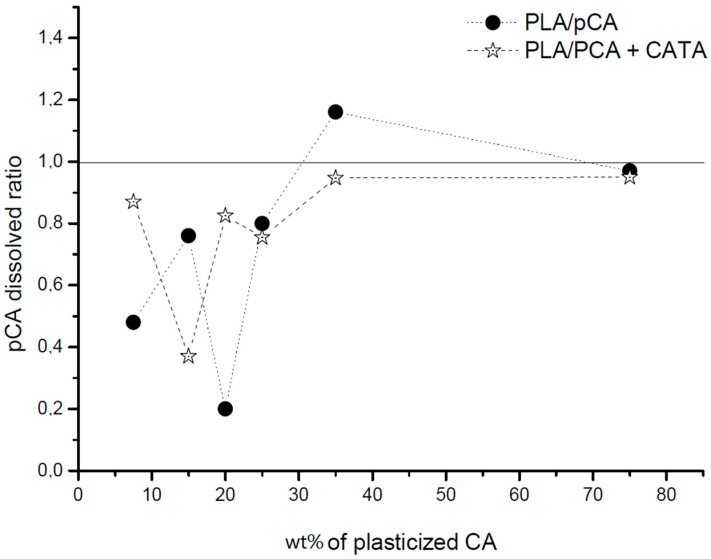
Dissolved cellulose acetate ratio as a function of the percentage of pCA in the blends.

The phase morphology of the blends prepared at 230 °C did not differ much from the blends prepared at 197 °C, as is evident in the morphology of the PLA75/pCA25 blend shown in Figure 11. However, especially in the uncatalysed blend, it is evident that the dispersed pCA phase was less fibrous than the corresponding blend prepared at 197 °C. The addition of the TBATPB catalyst resulted in a more fibrous morphology.

**Figure 12:**
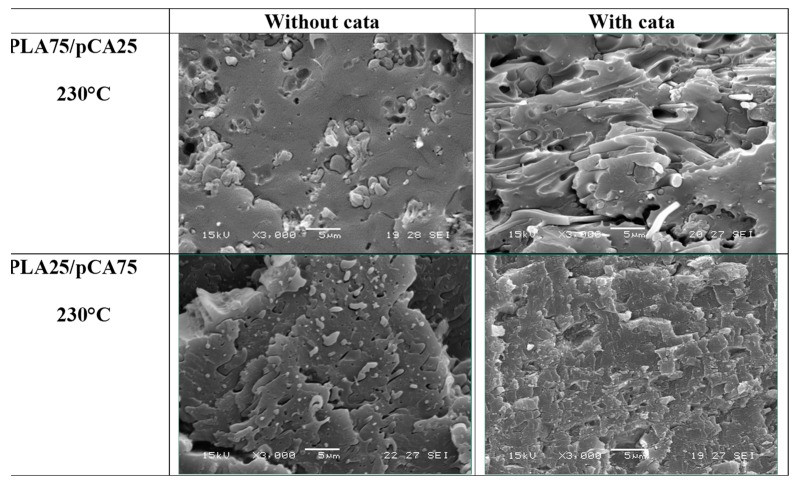
Scanning electron micrographs related to the PLA/pCA blends prepared at 230 °C with or without TBATPB.

The SEM analysis of the blends containing 75% of pCA showed a structure typical of a co-continuous distribution, in good agreement with the dissolution test results (Figure 12). Interestingly, the cryofractured surfaces of the blends obtained with the catalyst showed very high adhesion between the two phases and a low dimension of the phases, in agreement with an increased compatibility. On the other hand, considering the mechanical properties of these blends, it can be observed that the use of TBATPB resulted in a decrease in modulus, but in an increase in both tensile strength and elongation at break. These results can be attributed to the increased compatibility achieved thanks to the formation of low quantity PLA-CA copolymers during the rapid (60 s) reactive extrusion process [55]. A similar reaction also occurred in the blends with different compositions, but the evolution of morphology correlated to side reactions in the two phases did not provide evidence of any significant differences in properties between the compatibilised and uncompatibilised composites.

The correlation between impact properties and phase morphology as a function of PLA/pCA blends composition is summarised in Figure 6.

The analysis of the mechanical properties and the correlation with phase morphology shoed that the improvement of impact properties in the narrow composition range of 20–25% of pCA in both uncatalysed and catalysed blends is due to the formation of the fibrillated morphology that is typical of polymer composites. If we consider the blends with a PLA matrix and a pCA dispersed phase, the phase morphology, assuming that the system can be considered to consist of Newtonian droplets in a Newtonian matrix, is the result of different steps: the deformation, the break-up and the coalescence of the dispersed droplets [56]. By examining the results, it was possible to establish that the tendency to obtain a pCA fibrous structure can be related to the high value of the viscosity ratio in this system [56]. The high viscosity ratio favors the formation of fibrillated structures because it makes the break-up difficult. In the blends with a PLA matrix and a pCA dispersed phase, the viscosity ratio is high: at 230 °C, the torque ratio is 1.3, but at 197 °C, as this temperature is close to the melting peak of pCA, the CA phase is highly viscous, so the viscosity ratio is much higher.

It was noticed that the tendency to obtain a fibrous CA structure is more relevant at a lower temperature (197 °C vs. 230 °C). This can be attributed to the fact that the deformability, in simple shear context, is defined by Taylor as

(1) $$D_{\mathrm{Taylor}}=C_{a}\frac{16+19p}{16+16p}$$

where Ca is the capillary number and p is the viscosity ratio.

The capillary number is defined as

(2) $$C_{a}=\frac{\eta_{m}\cdot \dot{\gamma }\cdot R}{\Gamma }$$

where η_m_ is the viscosity of the matrix, $\dot{\gamma }$ is the shear rate and Γ is the interfacial tension. Hence, the deformability increases by increasing the viscosity of the matrix, and the torque of PLA matrix blends is almost doubled at 197 °C with respect to 230 °C (Table 1). Moreover, as previously discussed, the viscosity ratio is very high at 197 °C, as the pCA is very close to its melting temperature. Hence, the better properties of the blend extruded at 197 °C than at 230 °C can be mainly attributed to this difference in morphology of pCA in the PLA matrix

It can be also noticed that the tendency to obtain a fibrous structure seems slightly favored by the presence of TBATPB catalyst, especially at higher temperatures. This result can be attributed to the increased viscosity ratio due to the cross-linking of the pCA in the presence of TBATPB. Moreover, the TBATPB decreased the viscosity of the matrix because of PLA chain scission, discouraging the deformability of the pCA phase, but, on the other hand, the decrease in the interfacial tension (at the denominator in Equation (2)) due to the compatibility improvement, can counterbalance this effect. 

## 4. Conclusions

The extrusion of PLA with triacetine plasticized cellulose acetate (pCA), in the presence or absence of a transesterification catalyst, tetrabutyl ammonium tetraphenyl borate (TBATPB), was investigated with the aim of exploring the possibility of preparing PLA-based blends with an improved combination of stiffness and toughness.

It was shown, by torque analysis and specific TGA experiments, that the catalyst was responsible for darkening of the material as well as cross-linking of plasticized cellulose acetate. 

A range of compositions between 20 and 25% of pCA was found where blends exhibited improved impact properties with respect to pure PLA, without significantly affecting its elastic modulus and tensile strength. The properties were improved at 197 °C with respect to 230 °C. The analysis of phase morphology evolution as a function of composition showed that the observed improvement of properties can be explained by considering that pCA forms fibrillar particles in the PLA matrix during extrusion, especially at the lower temperature. An optimal dispersion of these fibres in the matrix was achieved for compositions of 20–25% of pCA. The blends thus consisted of composites with up to 25% of pCA, in which the PLA was the matrix and the cellulose acetate fibrillar particles were the reinforcing phase. The improvement of impact properties was thus attributed to both good adhesion between the two phases and the occurrence of pull-out during impact as mechanisms of energy dissipation that are typical of this class of composites.

The presence of the catalyst did not relevantly influence the trends in mechanical properties, but clear evidence of increased compatibility was found, especially in pCA-based blends. 

In general, the reactive extrusion of PLA in the presence of plasticized CA is a promising strategy for improving the PLA toughness without reducing its stiffness and can contribute to extending the use of this renewable and biodegradable polymer to applications where this combination of properties is necessary.

## Figures and Tables

**Table 1:** Polylactide/triacetine plasticized cellulose acetate (PLA/pCA) blends prepared at 230 °C and 197 °C and with triacetine and tetrabutylammonium tetra-phenylborate (TBATPB). N*cm = N·cm. “cata” indicate the trials where TBATPB was used as catalyst.

Samples	Extrusion Temperature T (°C)	TBATPB (wt%)	Triacetine Content (wt%)	Torque at 180 s (N*cm)
**PLA/pCA 75/25 230**	230	-	5	53 ± 12
**PLA/pCA 75/25 cata 230**	230	0.2	5	47 ± 4
**PLA/pCA 75/25 197**	197	-	5	99 ± 15
**PLA/pCA 75/25 cata 197**	197	0.2	5	90 ± 4

**Table 2:** Reference extrusion experiments carried out at 230 °C. pPLA = plasticized PLA; N*cm = N·cm.

Sample	Extrusion Temperature T (°C)	TBATPB (wt%)	Triacetine Content (wt%)	Torque at 180 s (N*cm)
**PLA**	230	-	-	47 ± 2
**pPLA**	230	-	5	37 ±1
**PLA cata**	230	0.2	-	41 ± 4
**pPLA cata**	230	0.2	5	33.7 ± 0.6
**pCA**	230	-	20	62 ± 10
**pCA cata**	230	0.2	20	80 ± 1

**Table 3:** Results of dissolution tests performed on extruded strands of blends.

Blend	PLA Content (%)	Processing Temperature (°C)	Residue Weight (%)
**AC7.5/PLA92.5**	92.5	197	96.4
**AC7.5/PLA92.5 + cata**	92.5	197	93.4
**AC15/PLA85**	85	197	88.6
**AC15/PLA85 + cata**	85	197	94.4
**AC20/PLA80**	80	197	96.0
**AC20/PLA80 + cata**	80	197	83.5
**AC25/PLA75**	75	197	80.3
**AC25/PLA75 + cata**	75	197	81.1
**AC35/PLA65**	65	197	59.4
**AC35/PLA65 + cata**	65	197	66.8
**AC75/PLA25**	25	230	27.5
**AC75/PLA25 + cata**	25	230	28.8
